# Response to the letter to editor regarding “finite element analysis of subtalar joint arthroereisis on adult acquired flexible flatfoot deformity using customized sinus tarsi implant”

**DOI:** 10.1016/j.jot.2022.12.002

**Published:** 2022-12-09

**Authors:** Duo Wai-Chi Wong, Yan Wang, Wenxin Niu, Ming Zhang

**Affiliations:** a Department of Biomedical Engineering, Faculty of Engineering, The Hong Kong Polytechnic University, Hong Kong, China; b The Hong Kong Polytechnic University Shenzhen Research Institute, Shenzhen, China; c Research Institute for Sports Science and Technology, The Hong Kong Polytechnic University, Hong Kong, China; d Shanghai YangZhi Rehabilitation Hospital (Shanghai Sunshine Rehabilitation Center), School of Medicine, Tongji University, Shanghai 201619, China

**Keywords:** Extra-osseous talotarsal stabilisation, Pes planus, Posterior tibial tendon dysfunction, Sinus tarsi implant, Talotarsal mechanism

## Dear editor

We thank Dr Chen and Dr Shi for their knowledgeable views and comments on our research of customised subtalar joint arthroereisis [1]. Dr Chen and Dr Shi expressed their opinions and concerns about whether the customised sinus tarsi implant could be appropriate to be regarded as “customised” if the sinus tarsi shape changes with foot positions.

We believe that “customisation” is a pragmatic procedure to consider individualised human factors, such as geometry, in design ergonomically. We do not view “customisation” as producing an exact geometrical match stringently, especially under a single static condition. In fact, the significance of customised design lies in its potential to accommodate the organic lifeform of Humans or other creatures. The scope and prospects of customisation would not be deterred by the dynamic nature. We have “customised” designs, from tailored suits to customised footwear and garment nowadays, that accommodate dynamic movement, clearance of the movement range, and topology of the Human body. With the advancement of 3D printing technology, we look forward to applying this concept to the medical field and further improving treatment outcomes for patients soon [2].

In medicine or healthcare, customisation often comes with rectification in both non-pathological and pathological conditions. For example, a pair of shoes customised with foot geometry needed to be modified with a toe spring and sufficient heel height to facilitate the push-off function and toe clearance. 3D-printed customised prostheses accounted for the unobserved geometry of the bone defect and the accommodation of revision surgery [3,4]. 3D printed orthotic foot insoles elevated the medial longitudinal geometry for therapeutic effects on flatfoot patients after shape customisation [5]. A recent publication on the customised total talar replacement kept abreast of the importance of joint motion, segment movements, and joint contact force during gait [6].

We agree with Dr Chen and Dr Shi that there was insufficient knowledge and much uncertainty on the customised design of the sinus tarsi implant for better clearance and functions. Customised designs shall have more considerations on the “dynamic shape” during functional motions, and “customisation” shall head from the patient-geometry-specific to the patient-movement-specific approach. A dynamic loading profile (with muscle forces) for subject-specific finite element models would be the challenge ahead [7]. While the subtalar arthroereisis procedure is an emerging procedure to treat hyperpronated flatfeet [8,9], our theoretical study with finite element analysis served as a starting point to explore the opportunities in customisation [1]. We hope our research could encourage and stimulate further evidence and design insights in rectifying customised implants to mitigate the threats of the procedure [10].

## Declaration of competing interest

The authors declare that they have no known competing financial interests or personal relationships that could have appeared to influence the work reported in this paper.
